# Inflammatory cytokines and plasma redox status responses in hypertensive subjects after heat exposure

**DOI:** 10.1590/1414-431X20155026

**Published:** 2016-02-02

**Authors:** S.F. Fonseca, V.A. Mendonça, M.C. Teles, V.G.C. Ribeiro, R. Tossige-Gomes, C.D.C. Neves, E. Rocha-Vieira, L.H.R. Leite, D.D. Soares, C.C. Coimbra, A.C.R. Lacerda

**Affiliations:** 1 Centro Integrado de Pós-Graduação e Pesquisa em Saúde, Universidade Federal dos Vales do Jequitinhonha e Mucuri, Diamantina, MG, Brasil; 2 Programa Multicêntrico de Pós Graduação em Ciências Fisiológicas, Sociedade Brasileira de Fisiologia, São Paulo, SP, Brasil; 3 Instituto de Ciências Biológicas, Universidade Federal de Juiz de Fora, Juiz de Fora, MG, Brasil; 4 Escola de Educação Física, Universidade Federal de Minas Gerais, Belo Horizonte, MG, Brasil; 5 Instituto de Ciências Biológicas, Universidade Federal de Minas Gerais, Belo Horizonte, MG, Brasil

**Keywords:** Hypertension, Heat, Cytokines, Redox status

## Abstract

Hypertension is characterized by a pro-inflammatory status, including redox imbalance and increased levels of pro-inflammatory cytokines, which may be exacerbated after heat exposure. However, the effects of heat exposure, specifically in individuals with inflammatory chronic diseases such as hypertension, are complex and not well understood. This study compared the effects of heat exposure on plasma cytokine levels and redox status parameters in 8 hypertensive (H) and 8 normotensive (N) subjects (age: 46.5±1.3 and 45.6±1.4 years old, body mass index: 25.8±0.8 and 25.6±0.6 kg/m^2^, mean arterial pressure: 98.0±2.8 and 86.0±2.3 mmHg, respectively). They remained at rest in a sitting position for 10 min in a thermoneutral environment (22°C) followed by 30 min in a heated environmental chamber (38°C and 60% relative humidity). Blood samples were collected before and after heat exposure. Plasma cytokine levels were measured using sandwich ELISA kits. Plasma redox status was determined by thiobarbituric acid reactive substances (TBARS) levels and ferric reducing ability of plasma (FRAP). Hypertensive subjects showed higher plasma levels of IL-10 at baseline (P<0.05), although levels of this cytokine were similar between groups after heat exposure. Moreover, after heat exposure, hypertensive individuals showed higher plasma levels of soluble TNF receptor (sTNFR1) and lower TBARS (P<0.01) and FRAP (P<0.05) levels. Controlled hypertensive subjects, who use angiotensin-converting-enzyme inhibitor (ACE inhibitors), present an anti-inflammatory status and balanced redox status. Nevertheless, exposure to a heat stress condition seems to cause an imbalance in the redox status and an unregulated inflammatory response.

## Introduction

Systemic arterial hypertension is characterized by endothelial dysfunction, vascular injury (1), and chronic inflammation (2), which includes increased pro-inflammatory cytokines such as interleukin 1-β (IL1-β), tumor necrosis factor (TNF-α) and interleukin 6 (IL-6). These cytokines affect the function of vascular endothelium-derived factors involved in blood pressure (BP) regulation (1,3,4). In addition, uncontrolled hypertension is associated with increased oxygen reactive species generation in multiple organs and tissues, including the brain, kidney and vasculature smooth muscle (5), which can lead to redox imbalance and consequent oxidative stress. Most clinical studies on hypertensive subjects found increased levels of thiobarbituric acid reactive substances (TBARS) in plasma, indicating augmented lipid peroxidation (6,7). Thus, cytokine release and oxidative stress formation are mechanisms that appear to be activated in hypertension (8).

Previous studies have demonstrated that heat stress exposure can increase both pro- and anti-inflammatory cytokines in human and animal models (9,10). Moreover, it seems that heat exposure may exacerbate oxidative stress (11) by uncoupling the mitochondrial respiratory chain or inhibiting antioxidant defense mechanisms (12). However, the effects of heat exposure in individuals with chronic inflammatory diseases, such as hypertension, are complex and not completely understood.

To date, no study has analyzed the inflammatory consequences of heat exposure in hypertension. Performing daily living activities in hot environments has gained attention in recent decades because of the progressive increases in environmental temperature, which is directly related to hospitalizations for cardiovascular disease (13). Taking into account that hypertensive subjects have chronic inflammation, which is associated with development of elevated blood pressure and future risk of myocardial infarction, atherosclerosis and cardiovascular death (8,14,15), and that heat exposure interferes with inflammatory and redox status, this study aimed to investigate the effects of heat exposure on plasma cytokine levels and redox status parameters in hypertensive subjects.

## Material and Methods

### Ethical statement

This study was conducted in accordance with the ethical principles for research involving humans (Resolution 196-96 of the National Health Council of the Brazilian Ministry of Health) and was approved by the Universidade Federal dos Vales do Jequitinhonha e Mucuri Ethics Committee (protocol #024/12). All participants were informed about the study procedures and provided written consent to participate in this study.

### Subjects

Eight male subjects with controlled essential hypertension were taking antihypertensive medication (angiotensin-converting-enzyme inhibitor (ACE inhibitors) and diuretics) and 8 normotensive subjects matched according to age, weight, height and ethnicity volunteered to participate in this study. All volunteers were non-smokers and non-obese and were not heavy alcohol consumers.

### Preliminary procedures

A preliminary assessment was performed to determine individual body composition by skin fold measurements (16). The results were used to obtain the body fat percentage (17). Body mass index was calculated by Quetelet's equation (18) from measured weight and height.

BP was measured in both groups (hypertensive and normotensive) during 5 consecutive days in the morning at each volunteer's home, after 10 min of rest in a sitting position.

### Experimental protocol

Individuals underwent a fast for 8 h before the experimental procedures to eliminate any food intake influence. On the day of the assessment, each subject remained at rest in a sitting position for 10 min in a thermoneutral environment (22°C) and then spent 30 min in an environmental chamber with a controlled 38°C dry bulb temperature with 60% relative humidity (∼32°C of the wet bulb globe temperature). Heart rate (HR) was recorded in the thermoneutral environment and after exposure to heat by a telemetric HR monitor (POLAR RS800sd, Polar, Finland), and BP was measured using an inflated cuff mercury sphygmomanometer and stethoscope. The systolic and diastolic pressures were registered at the first and fourth Korotkoff sound, respectively. Additionally, blood samples were collected before and after heat exposure. All experimental procedures were performed during the morning.

The specific gravity (Ug) of urine was determined to assess the subject's hydration status with the use of a portable hand-held refractometer (model 301, Biobrix, Brazil) calibrated with distilled water. Subjects were considered hydrated when the Ug <1.030 (19). The volunteers collected urine 5 min before beginning the experimental protocol.

### Cytokine measurement

For plasma processing, 9 mL of whole blood was collected from the antecubital vein using aseptic techniques and heparin as an anticoagulant. The blood was then centrifuged, and the plasma was stored at -80°C. High sensitivity sandwich ELISA kits (R&D Systems, USA) were used to determine interleukin 10 (IL-10), IL-6, TNF-α, and IL1-β plasma levels. Soluble TNF receptor (sTNFR1, sTNFR2) levels were measured using conventional sandwich ELISA kits (DuoSet, R&D Systems). Procedures were conducted according to the manufacturer's instructions. The detection limits were 0.10 pg/mL for IL-6, IL-10, TNF-α, and IL1-β and 12 pg/mL for both soluble receptors.

### Redox status measurement

For plasma processing, 6 mL of whole blood was collected from the antecubital vein using aseptic techniques and ethylenediamine tetra-acetic acid (EDTA) as an anticoagulant. Blood redox status was assessed by TBARS and ferric reducing ability of plasma (FRAP) determination, as described elsewhere (20,21).

As an index of lipid peroxidation, we used the formation of TBARS during an acid-heating reaction (20). Briefly, 0.4 mL of sample was added to 0.25 mL of acetic acid (2.5 M, pH 3.4), and 0.25 mL of 0.8% thiobarbituric acid (Sigma, USA), and they were then heated in a boiling water bath (90°C) for 90 min. After the addition of n-butanol, samples were centrifuged (10 min, 150 *g*), the butanol layer was collected and TBARS were determined by the absorbance at 532 nm (Spectra Max 190, Molecular Devices, USA), compared with a standard curve constructed with known concentrations of malondialdehyde (MDA)(1,1,3,3-tetramethoxypropane; Sigma) as an external standard. The amount of MDA produced was interpreted as the TBARS levels and indicates the degree of lipid peroxidation. The results are reported as MDA equivalents per mg protein. Measurements were performed in duplicates.

Plasma antioxidant status was evaluated with the FRAP assay (21), based on the reduction of ferric-tripyridyltriazine [Fe(III)-TPTZ] complex to ferrous tripyridyltriazine [Fe(II)-TPTZ] at a low pH, by antioxidants present on plasma, resulting in a color change that was measured by absorbance at 550 nm. Plasma samples of 0.15 µL were mixed with 264 µL of pre-warmed (37°C) freshly prepared FRAP reagent [10 mM TPTZ (2,4,6-tripyridyltriazine), 20 mM FeCl_3_, 0.3 mol/L sodium acetate buffer, pH 3.6] and incubated for 15 min, 37°C. Absorbance at 595 nm was determined, using a plate reader (Spectra Max 190, Molecular Devices) and FRAP estimated by comparison with a standard curve constructed with known concentrations of FeSO_4_. The results are reported as FeII equivalents per mg protein. Measurements were performed in duplicate.

### Statistical analysis

Data are reported as means±SE. The normality of the variables was evaluated with the Shapiro-Wilk normality test. Log-transformations were used to normalize the cytokine data and redox status biomarkers, excluding the soluble TNF receptors, which were normally distributed. The Student's unpaired *t*-test was used only to assess differences between means of the subject characteristics (Table 1). Student's paired *t*-test was used in the normotensive or hypertensive groups separately in order to evaluate the effect of heat exposure in the inflammatory and redox status. Two-way ANOVA with a Bonferroni *post hoc* test was used to assess the disease effect, the heat effect, and the interactions (group *vs* environment). The significance level for all tests was P<0.05.

**Table:**
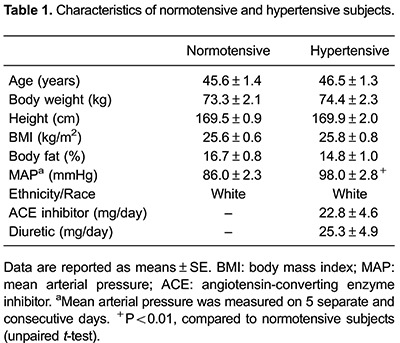

## Results

There were no significant differences between hypertensive and normotensive subjects for weight, height, age, BMI, and body fat percentage.

Hypertensive individuals were taking diuretics and ACE inhibitors at the time of the study. Despite the use of the drug combination, the mean arterial pressure (MAP) of these subjects was higher compared to the normotensive subjects. Nevertheless, the BP values of hypertensive subjects were within the physiologically acceptable range (22) (Table 1).

**Figure 1:**
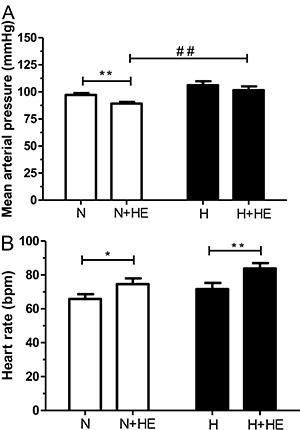
Effect of heat exposure (+HE) on mean arterial pressure (*A*) and heart rate (*B*) in normotensive (N) and hypertensive (H) subjects. Data are reported as means±SE. *P<0.05 or **P<0.01 in intra-group comparisons (Student's paired *t*-test). ^##^P<0.01 in inter-group comparisons (two-way ANOVA: group P<0.01; time P<0.05; interaction P=0.56).

The MAP and HR did not differ between groups in the thermoneutral environment. However, heat exposure decreased the mean arterial pressure in normotensive subjects (P<0.01) and was significantly lower compared to hypertensive subjects (P<0.01). Heat exposure also increased HR in both groups in a similar way (N: P<0.05 and H: P<0.01; Figure 1).

**Figure 2:**
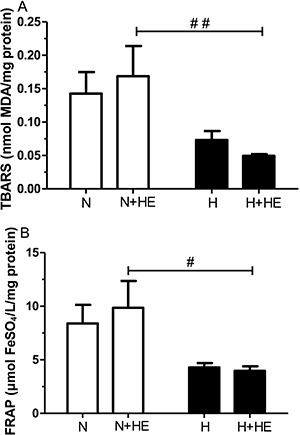
Effect of heat exposure (+HE) on the plasma levels of thiobarbituric acid reactive substances (TBARS) (*A*) and ferric reducing ability of plasma (FRAP) (*B*) in normotensive (N) and hypertensive (H) subjects. ^##^P<0.01 inter-group comparisons for TBARS (two way ANOVA: group P<0.01; time P=0.54; interaction P=0.37) and ^#^P<0.05 inter-group comparisons for FRAP (two-way ANOVA: group P<0.05; time P=0.95; interaction P=0.66).

For plasma redox status, there was no difference in TBARS and FRAP (Figure 2) between normotensive and hypertensive individuals. However, after heat exposure, both TBARS levels and FRAP were lower in hypertensive subjects compared to normotensive subjects.

**Figure 3:**
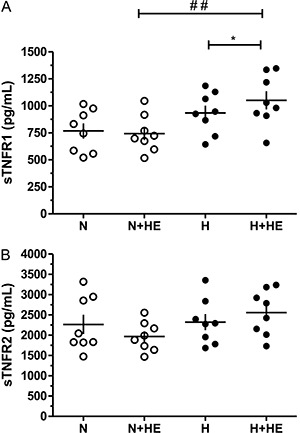
Effect of heat exposure (+HE) on the plasma levels of soluble TNF receptor sTNFR1 (*A*) and sTNFR2 (*B*) in normotensive (N) and hypertensive (H) subjects. Data are reported as means±SE. *P<0.05 in intra-group comparisons (Student's paired t-test). ^##^P<0.01 inter-group comparison (two-way ANOVA: group P<0.01; time P=0.53; interaction P=0.33).

Figure 3 shows the plasma levels of sTNFR1 (*A*) and sTNFR2 (*B*) in normotensive and hypertensive subjects in response to heat exposure. No differences were observed between groups in the thermoneutral environment. However, there was an increase in the sTNFR1 plasma level in hypertensive subjects (P<0.01) after heat exposure, which was significantly higher compared to the sTNFR1 level in normotensive subjects (P<0.05).

**Figure 4:**
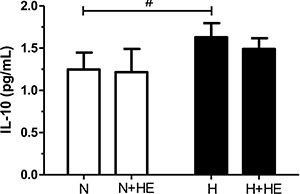
Effect of heat exposure (+HE) on the plasma levels of interleukin 10 (IL-10) in normotensive (N) and hypertensive (H) subjects. Data are reported as means±SE. ^#^P<0.05 inter-group comparison (two-way ANOVA: group P<0.05; time P=0.44; interaction P=0.70).

The hypertensive subjects had significantly higher IL-10 plasma levels in the thermoneutral environment (Figure 4; P<0.05). However, heat exposure minimized this effect and resulted in no difference between groups.

The calculation of statistical power for the sTNFR1 (the strongest predictor of survival in a panel of pro-inflammatory markers in inflammatory chronic diseases) (23,24) and IL-10 (potent anti-inflammatory, which suppress the expression of many inflammatory cytokines, including TNFα, IL-6 and IL-β) (14,25 [Bibr B26]), considering an F of 0.65 and 0.56, respectively (alpha value=0.5) and a sample size of 8 subjects, revealed a statistical power above 77% for the sTNFR1 and a statistical power above 60% for the IL-10.

There were no significant differences in the plasma levels of the pro-inflammatory cytokines, IL-6, IL-1β and TNF-α in the thermoneutral environment or during heat exposure (Table 2).

**Table:**
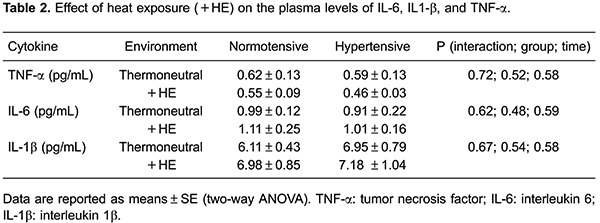

All subjects were hydrated and had similar urine specific gravity (N: 1011±1.99 Ug; H: 1015±2:06 Ug; P=0.28).

## Discussion

Understanding the effects of heat exposure on inflammatory and redox status parameters in hypertensive subjects is important, considering that 1) a large number of people are exposed to hot environmental conditions in their daily lives; 2) some organizations have issued warnings about climate change with the prospect of further increases in both global average temperature and the frequency of heat waves (26); 3) these changes may have a direct impact on population health, especially in subjects with systemic arterial hypertension who have a higher risk of developing heat-related illness (27); and 4) inflammatory changes may often be present before clinical symptoms and structural changes occur (23).

In the present study, the MAP of normotensive subjects was reduced after heat exposure and was consequently lower compared to hypertensive subjects; while heat exposure increased HR in both groups in a similar way. These findings were not surprising because heat stress exposure triggers cardiovascular adjustments to maintain thermal homeostasis and ensure adequate cardiac output for maintaining organs and systems (28).

The main findings of this study were that hypertensive subjects had significantly higher IL-10 plasma levels, an anti-inflammatory cytokine, and that after heat exposure, IL-10 levels were not different compared to those of normotensive subjects. Furthermore, heat exposure increased sTNFR1 plasma levels in hypertensive subjects compared to normotensive subjects and reduced both TBARS and FRAP in hypertensive subjects.

Following heat exposure, hypertensive individuals had higher plasma levels of sTNFR1, which suggests an increased pro-inflammatory status by exposure to heat in these individuals, although this effect was not observed in the pro-inflammatory cytokine TNF-α itself. This finding may be related to the instability of this cytokine, which has a short half-life that can vary throughout the day (29). The biological activity of TNF-α is mediated by its membrane receptors, TNFR1 (p55) and TNFR2 (p75) (30,31). Both receptors are released in soluble form (sTNFR1 and sTNFR2) by proteolytic cleavage of the forms associated with the surface membrane, exerting an important role in regulating TNF-α activity. The sTNFRs can inhibit the effects of TNF-α by competing with membrane receptors, serve as carriers of TNF-α and, in some cases, increase their effects. In this case, sTNFRs prolong their function and are considered slow-release reservoirs. Due to difficulty detecting them in plasma, usually because of low levels of TNF-α, it has been suggested to measure their soluble receptors (30). TNFR1 is associated with a pro-inflammatory response and apoptosis, while TNFR2 is associated with tissue repair and angiogenesis (32). According to these findings, Rauchhaus et al. (24) and Von Haehling et al. (23) suggested that the sTNFR1 may be the strongest predictor of survival in a panel of pro-inflammatory markers in inflammatory chronic diseases.

Unlike the results of this study, previous research has demonstrated increased plasma levels of pro-inflammatory cytokines, including TNF-α and IL-6, in non-treated hypertensive patients compared to control patients (33). However, in this study, H subjects were taking ACE inhibitors and diuretics for more than 12 months and, thus, had controlled BP. Therefore, because previous studies reported that ACE inhibitors can reduce IL-6 and IL1-β in hypertensive animals and humans (34,35) and increase levels of IL-10 after 12 weeks of treatment (34), it seems reasonable to believe that the use of ACE inhibitors by hypertensive subjects in our study may have influenced their IL-10 plasma levels. It is worth mentioning that IL-10 has potent anti-inflammatory properties, which repress expression of many inflammatory cytokines, including TNFα, IL-6 (14) and IL1-β (25). Furthermore, the increase in this anti-inflammatory cytokine may exert protective effects in subjects with hypertension. Because the present study showed controlled BP in hypertensive subjects with the use of drugs, it is believed that there may have been an influence of the drug on the production of the IL-10 anti-inflammatory cytokine (36). However, because heat exposure minimized this effect and resulted in no difference between groups, it is possible that the environmental heat stress suppressed this effect.

The use of ACE inhibitors to control BP may also have influenced the redox status parameters evaluated in this study taking into account that previous studies have demonstrated the beneficial effect of chronic use of ACE inhibitors on vascular oxidative stress and endothelial dysfunction in hypertensive subjects (37,38). Thus, the use of antihypertensive drugs may have contributed to the antioxidant condition characterized by lower TBARS levels observed in hypertensive subjects compared to normotensive subjects in the heat stress condition. Moreover, the redox status was determined by lower levels of TBARS and FRAP in hypertensive subjects compared to normotensive subjects under heat stress. It may well be that the lower FRAP seen in hypertensive subjects after heat exposure is the consequence of a balance between oxidants and antioxidants, also followed by ACE inhibitors-induced lower production of oxidants (TBARS).

Briefly, angiotensin II is considered a pivotal player in the altered central and peripheral redox status in hypertension. It also triggers inflammation and other metabolic responses (39). On the contrary, the use of ACE inhibitors as antihypertensive therapy can improve inflammatory and redox status (37,38). However, it is supposed that these beneficial effects of chronic use of ACE inhibitors are minimized or suppressed by heat stress.

Finally, since excessive perception of their own or other people's emotions can lead to increased clinical and ambulatory blood pressure levels (40), the measurement of emotional/psychological status should be regarded in future studies. Moreover, the sample size might be considered a potential limitation of the present study because different conditions in hypertensive subjects (such as obesity and others) could potentially produce variations in terms of inflammatory and redox status parameters in different studies. Nevertheless, the consistency of the present results for all patients is enough to support our conclusions. Furthermore, the results of this study provide subsidies for understanding the plasma cytokine levels and redox status parameters after heat exposure in subjects who use anti-hypertensive medication, a common situation found in everyday life of the hypertensive population.

In conclusion, the present study showed that controlled hypertensive subjects taking ACE inhibitors presented an anti-inflammatory status and a balanced redox status. However, exposure to a heat stress condition seemed to cause an imbalance in the redox status and an unregulated inflammatory response.
